# Associations of Genetic Variations in *ABCA1* and Lifestyle Factors with Coronary Artery Disease in a Southern Chinese Population with Dyslipidemia: A Nested Case-Control Study

**DOI:** 10.3390/ijerph16050786

**Published:** 2019-03-04

**Authors:** Tian-Yu Zhao, Song Lei, Liu Huang, Yi-Nan Wang, Xiao-Ni Wang, Ping-Pu Zhou, Xiao-Jun Xu, Long Zhang, Liang-Wen Xu, Lei Yang

**Affiliations:** 1 Medical School, Hangzhou Normal University, Hangzhou 310000, China; zhaotianyu0225@foxmail.com (T.-Y.Z.); leisong713@gmail.com (S.L.); huangliu_mark@163.com (L.H.); wangyn60232@163.com (Y.-N.W.); wxn451hhh@163.com (X.-N.W.); zpingp0911@163.com (P.-P.Z.); Xiaojunxu0202@163.com (X.-J.X.); zhanglongm@foxmail.com (L.Z.); lwxu2006@163.com (L.-W.X.); 2 Medical School, Shihezi University, Shihezi 832000, China

**Keywords:** *ABCA1*, lifestyle, coronary artery disease, interaction, haplotype

## Abstract

Coronary artery disease has become a major health concern over the past several decades. We aimed to explore the association of single nucleotide polymorphisms (SNPs) in the ATP-binding cassette subfamily A member 1 (*ABCA1*) and lifestyle factors with coronary artery disease (CAD) in dyslipidemia. This nested case-control study included 173 patients with CAD and 500 matched control individuals (1:3, case: control) from a district in southern China. We collected medical reports, lifestyle details, and blood samples of individuals with dyslipidemia and used the polymerase chain reaction-ligase detection reaction method to genotype the SNPs. The CC genotype of the additive and recessive models of rs4149339, together with regular intake of fried foods or dessert, increased the risk of CAD (adjusted odd ratio (OR) = 1.91, *p* = 0.030; adjusted OR = 1.97, *p* = 0.017; adjusted OR = 1.80, *p* = 0.002; adjusted OR = 1.98, *p* = 0.001). The AT + AA genotype of the dominant model of rs4743763 and moderate/heavy physical activity reduced the risk of CAD (adjusted OR = 0.66, *p* = 0.030; adjusted OR = 0.44, *p* = 0.001). The CT + CC genotype of the dominant model of rs2472386 reduced the risk of CAD only in males (adjusted OR = 0.36, *p* = 0.001). The interaction between rs4149339 and rs4743763 of *ABCA1* and haplotype CTT (comprising rs4149339, rs4743763, and rs2472386) appeared to increase the risk of CAD (relative excess risk due to interaction (RERI) = 3.19, *p* = 0.045; OR = 1.49, *p* = 0.019). Polymorphisms of rs4149339, rs4743763 and rs2472386 in *ABCA1* and three lifestyle factors (physical activity, fried food intake, and dessert intake) were associated with CAD in people with dyslipidemia in southern China. These results provide the theoretical basis for gene screening and the prevention of chronic cardiovascular diseases.

## 1. Introduction

In recent years, cardiovascular and cerebrovascular diseases, represented by coronary artery disease and stroke, have become the world’s top causes of mortality in humans [1]. According to the 2017 China Cardiovascular Disease Report, 11 million people in urban and rural areas suffer from coronary artery disease. The mortality rate of patients with coronary artery disease increased from 39.56 per 100,000 in 2002 to 110.91 per 100,000 in 2015, an increase of nearly three times [2]. Atherosclerosis is the main basis for the onset of coronary heart disease and it usually manifests as vascular endothelial cell damage and endothelial function decline caused by dyslipidemia, which are chronic inflammatory responses [3]. Dyslipidemia and atherosclerosis have been assessed by a large number of epidemiological studies [4,5,6,7]. Compared with individuals with normal blood lipid levels, individuals with abnormal lipid levels are more likely to develop atherosclerotic lesions, eventually leading to severe cardiovascular diseases such as coronary artery disease [8,9]. 

Coronary artery disease is a complex disease that is affected by genetic and environmental factors. Most genes can affect lipid metabolism in the body, followed by the development of atherosclerosis [10,11,12,13,14,15,16]. Some studies have found that cardiovascular diseases such as coronary heart disease have a strong genetic basis, demonstrated by family associations and degrees of genetic influence as high as 40–50% [17,18]. ATP-binding cassette transporter A1 (*ABCA1*) is a member of the ATP-binding cassette transport factor gene family, whose main function is to balance the cholesterol concentration inside and outside the cell to maintain normal levels among various blood lipids. It is also involved in the formation of the atherosclerotic inflammatory response, which may be directly related to the occurrence and development of atherosclerotic lesions [19]. In addition, lifestyle factors, such as lack of physical activity and a regular intake of fried foods and sweets, are also suggested to be risk factors for coronary artery disease (CAD) [20,21,22]. 

The present study was a community-based nested case-control study to analyze the association of three *ABCA1* single nucleotide polymorphisms (SNPs, one in the 3’-untranslated region and two in the intron region) and certain lifestyle factors (dessert and fried food intake and physical activity intensity) with CAD in a Han Chinese dyslipidemic population in southern China.

## 2. Methods 

### 2.1. Subjects

We randomly selected four townships under the jurisdiction of Ningbo, Zhejiang Province, as survey points by cluster random sampling. A total of 2349 people who underwent physical examinations at the community health service center from April 2013 to July 2013 and had dyslipidemia were included. All subjects were unrelated and >40 years of age. We excluded patients treated with antihypertensive drugs or hypolipidemic drugs, as well as patients with severe liver and kidney disease and malignant tumors. Lifestyle information and blood samples were collected from all subjects, and a visit was conducted in August 2016 to obtain data on the incidence of CAD in the subjects. The case group included 173 patients diagnosed with CAD between April 2013 and August 2016. Five hundred age- and sex-matched subjects were included as control individuals (1:3, case: control). All participants signed informed consent forms. The research protocol was approved by the Medical Ethics Committee of the Affiliated Hospital of Hangzhou Normal University.

### 2.2. Diagnostic Criteria

Dyslipidemia was defined according to the Guidelines for the Prevention and Treatment of Diabetes in Chinese Adults prepared by the Joint Committee of the Chinese Association for the Prevention and Treatment of Dyslipidemia. Total cholesterol (TC) > 5.18 mmol/L, triglycerides (TG) > 1.70 mmol/L, low-density lipoprotein cholesterol (LDL-C) > 3.37 mmol/L, and high-density lipoprotein cholesterol (HDL-C) < 1.04 mmol/L represent abnormal values. One or more of the above four blood lipid indicators suggest a diagnosis of dyslipidemia. Subjects diagnosed with dyslipidemia and taking hypolipidemic drugs were also considered under the dyslipidemia category. CAD is usually diagnosed by computed tomography, radiography, or coronary angiography. The diagnosis was based on the diagnostic criteria for coronary atherosclerotic heart disease issued by the China Health and Family Planning Commission in 2010 [23]. Clinically diagnosed coronary artery ischemia, asymptomatic coronary heart disease, myocardial infarction, angina pectoris, or ischemic cardiomyopathy are all covered under CAD. 

### 2.3. Epidemiological Investigation

The field epidemiological investigation mainly included basic demographic criteria such as age, sex, occupation, and education level, as well as information on lifestyle such as diet and intensity of physical activity. Among them, the dietary behavior survey employed a semi-quantitative questionnaire, divided into food type, intake, and frequency. The main variables of lifestyle were defined as follows. (1) Diet: average weekly intake of fried food less than once was defined as “no fried food intake,” and average weekly dessert intake less than once was defined as “no dessert intake.” (2) Physical activity classification: “secretarial work, office work, etc.” was defined as “sedentary physical activity”; “sales, hotel service, chemical experimentation, lecturing, etc.” was defined as “light physical activity”; “daily student activities, motor vehicle driving, electrician work, metalworking, cutting, etc.” was defined as “moderate physical activity”; and “steelmaking, dance, sports, loading and unloading goods, construction work, etc.” was defined as “heavy physical activity.” Anthropometric data, including waist circumference; body mass index (BMI); systolic blood pressure (SBP); diastolic blood pressure (DBP); and TC, TG, HDL-C, and LDL-C levels, were evaluated by professional medical examination according to standard protocols.

### 2.4. SNP Selection and Genotyping

Three SNPs in *ABCA1* were selected using the HapMap website and the Haploview 4.2 software (Broad Institute, Cambridge, MA, USA). The SNP rs4149339 resides in the 3′-UTR of *ABCA1*, and rs4743763 and rs2472386 reside in the intron of *ABCA1*. The minor allele frequencies (MAFs) of these three SNPs were greater than 5%, and the linkage disequilibrium r2 > 0.8.

Five milliliters of whole blood from each fasted individual was anticoagulated with EDTA and stored in a refrigerator at −80 °C. DNA was extracted using Tiangen Blood Genomic DNA extraction kits (Tiangen Biotech, Beijing, China) and sent to Shanghai Jierui Biological Engineering Co., Ltd., for genotyping analysis using the polymerase chain reaction (PCR)-ligase detection reaction (LDR) method (Generay Biotech Company, Shanghai, China). The primer sequences of rs4149339 were 5′-TCTTGGCTTTTGCATTGTTG-3′ (forward) and 5′-CTGTGCCATGTTATTCAGCTC-3′ (reverse). The primer sequences of rs4743763 were 5′-TCTGTCATGTGGCTGCAACT-3′ (forward) and 5′-ATGCAACAGATGCCCTATCC-3′ (reverse). The primer sequences of rs2472386 were 5′-TTCCCCTGCATCAAGTTTTC-3′ (forward) and 5′-TTGATCTGCCCTTTGTTTCC-3′ (reverse). The PCR reaction volume contained 1 µL DNA, 1.5 µL 10X buffer, 1.5 µL MgCl_2_, 0.3 µL dNTPs, 0.15 μL of each primer, 0.2 µL Taq enzyme, and water to make the total volume 15 µl. The amplification conditions were as follows: 94 °C for 3 min; 35 cycles of denaturation at 94 °C for 15 s, annealing at 55 °C for 15 s, and extension at 72 °C for 30 s; and 72 °C for 3 min. The ligation reaction volume comprised 3 µL PCR product, 1 µL 10X Taq DNA ligase buffer, 0.125 µL Taq DNA ligase (40 U/µl), 0.01 μL of each discriminating probe, and water to make the total volume 10 µL. The reaction conditions were as follows: 30 cycles of 94 °C for 30 s and 56 °C for 3 min. To 1 µL of extension product, 8 µL of loading buffer was added, followed by denaturation at 95 °C for 3 min. Next, the product was immediately bathed in ice water. The mixture was analyzed using a sequencer (ABI 3730XL). For quality control, we randomly chose 10% of samples for re-genotyping, and the concordance was 100%.

### 2.5. Statistical Analysis

Statistical analysis was performed using SPSS 24.0 software (SPSS Inc., Chicago, IL, USA). Demographic characteristics and the SNP genotypes of *ABCA1* were evaluated using the χ^2^ test, Fisher’s exact test (for categorical variables), Student’s *t*-test, and Wilcoxon’s rank sum test (for continuous variables). The χ^2^ test was used to test for Hardy–Weinberg equilibrium. The associations of genetic models and lifestyle factors with the risk of CAD were estimated by calculating odd ratios (ORs) and 95% confidence intervals (95% CIs) using logistic regression analysis. The forest map used R language (package “forestplot”). The relative excess risk due to interaction (RERI), OR, and 95% CI were determined using Microsoft Excel according to Knol et al. [24]. The associations between *ABCA1* haplotypes and the risk of CAD were calculated using the R language package “haplo.stats.” In all analyses, *p* values less than 0.05 were considered statistically significant.

## 3. Results

### 3.1. Basic Characteristics, Lifestyle Factors, and Genotype Distribution in Case and Control Groups

The subjects included 173 cases and 500 controls; 42.3% of the subjects were female, and 57.7% were male. The mean BMI was 24.23 ± 3.23 kg/m^2^ in case subjects, which was significantly more than that in control subjects (23.11 ± 2.93 kg/m^2^) (*p* < 0.001). The medians of waist circumference, DBP, and LDL-C in the case group were significantly higher than those in the control group (*p* < 0.05) (Table 1).

All three of the studied SNPs in the control subjects were in Hardy–Weinberg equilibrium (*p* > 0.05) (Table S1). Linkage disequilibrium (LD) analysis for the three SNPs showed obvious LD between two SNPs (Table S2). The χ^2^ test found significant differences for physical activity, fried food intake, dessert intake, and the rs4149339 genotype between the case group and the control group (*p* < 0.05) *(*Table 1*).*

Stratified by sex, in the case group and control group, HDL-C, physical activity, derssert intake, fried food intake, and rs4743763 and rs2472386 genotypes were found to be statistically significant only in male subjects. The rs4149339 genotype was found to be statistically significant only in female subjects (Table S3). The other meaningful results are the same as in the previous table.

### 3.2. Associations of Genetic Models and Lifestyle Factors with the Risk of CAD

**Figure 1:**
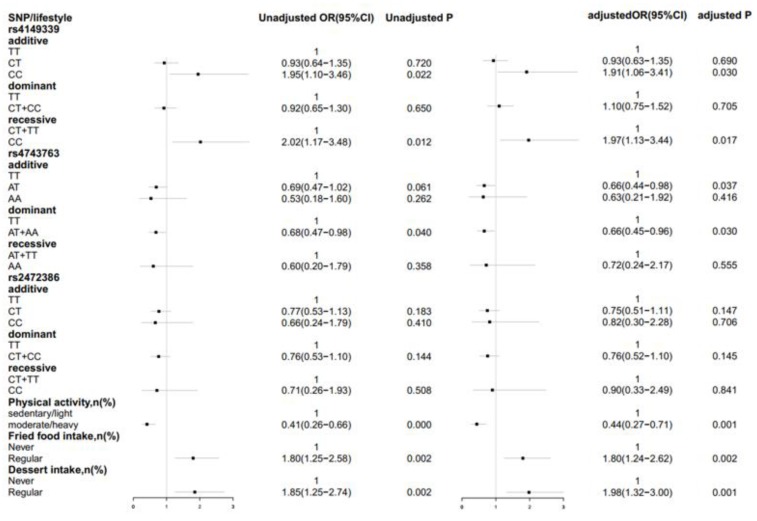
Forest map for the risk of coronary artery disease (CAD) with different genetic models and lifestyle factors. Adjusted for age, sex, waist circumference, smoking, and drinking. *p* value < 0.05 was considered statistically significant and maintains significance using the Benjamini-Hochberg procedure with the false discovery rate at 0.11.

The association between the *ABCA1* gene and lifestyle factors with CAD in dyslipidemia was examined under each gene model. With or without adjustment for the confounding factors age, sex, waist circumference, smoking, and drinking, the rs4149339 additive and recessive models, the rs4743763 dominant model, physical activity, fried food intake, and dessert intake were found to be significantly associated with CAD in dyslipidemia. In rs4149339 additive model, people carrying the CC genotype were found to be 0.91 times more likely to develop CAD than those with the TT genotype (adjusted OR = 1.91, 95% CI = 1.06–3.41, *p* = 0.030). People carrying the CC genotype of the recessive model were at a higher risk of CAD than those with the TT + CT genotype (adjusted OR = 1.97, 95% CI = 1.13–3.44, *p* = 0.017). People carrying the AT + AA genotype of the dominant model of rs4743763 had a lower risk of CAD than those with the TT genotype (adjusted OR = 0.66, 95% CI = 0.45–0.96, *p* = 0.030). Compared with people performing sedentary/light physical activity, those performing moderate/heavy physical activity were less susceptible to CAD (adjusted: OR = 0.44, 95% CI = 0.27–0.71, *p* = 0.001). Regular intake of fried foods was 0.80 times more likely to cause CAD in dyslipidemia patients than in those who did not consume fried foods (adjusted: OR = 1.80, 95% CI = 1.24–2.61, *p* = 0.002). Similarly, regular intake of dessert posed a 0.98 times higher risk of CAD compared to no dessert intake (adjusted: OR = 1.98, 95% CI = 1.32–3.00, *p* = 0.001) (Figure 1).

After stratification by sex, we found that carrying the AT + AA genotype of dominant model rs4743763 (adjusted OR = 0.28 , 95% CI = 0.15–0.55, *p* < 0.001), the CT+CC genotype of dominant model of rs2472386(adjusted OR = 0.36, 95% CI = 0.19–0.67, *p* = 0.001) and moderate/heavy physical activity(adjusted OR = 0.24, 95% CI = 0.11–0.51, *p* < 0.001) are protective factors for CAD in males (Table S4). Regular Fried food intake (adjusted: OR = 2.48, 95% CI = 1.37–4.51, *p* = 0.003) and regular dessert intake (adjusted: OR = 2.69, 95% CI = 1.41–5.12, *p* = 0.003) are risk factors for CAD in males. rs4149339 CC genotype of recessive model (adjusted: OR = 2.11, 95% CI = 1.04–4.25, *p* = 0.037) is protective factor for CAD in females (Table S5).

### 3.3. Interactions of ABCA1 SNPs and Lifestyle Factors with CAD in Dyslipidemia

Table 2 shows the effect of the interaction of the *ABCA1* rs4149339 polymorphism and lifestyle factors on coronary artery disease. After adjustment for age, sex, waist circumference, smoking, and drinking, compared with people carrying the CT + TT genotype who performed moderate/heavy physical activity, those with the CT + TT or CC genotype who performed sedentary/light physical activity were at a higher risk of CAD (OR = 2.45, 95% CI = 1.46–4.13, *p* = 0.001; OR = 4.10, 95% CI = 1.94–8.67, *p* < 0.001). Compared with people carrying the CT + TT genotype and having no fried food intake, those with the CT + TT or CC genotype and having regular fried food intake were at an increased risk of CAD (OR = 1.88, 95% CI = 1.26–2.79, *p* = 0.003; OR = 2.98, 95% CI = 1.47–6.02, *p* = 0.002). People carrying the CC genotype with or without dessert intake and those carrying the CT + TT genotype and with regular dessert intake were at a higher risk of CAD than individuals carrying the CT + TT genotype and having no dessert intake (OR = 5.26, 95% CI = 1.81–15.25, *p* = 0.002; OR = 3.04, 95% CI = 1.46–6.34, *p* = 0.003; OR = 2.29, 95% CI = 1.47–3.56, *p* < 0.001). The interaction between the rs4149339 polymorphism and lifestyle factors was not found in the additive model (*p* values of RERI > 0.05).

Table 3 shows the effects of the interaction between the *ABCA1* rs4743763 polymorphism and lifestyle factors on coronary artery disease. After adjustment for age, sex, waist circumference, smoking, and drinking, compared with people carrying the AT + AA genotype who performed moderate/heavy physical activity, those with the AT + AA genotype who performed sedentary/light activity, as well as those with the TT genotype and performing sedentary/light physical activity had a higher risk of CAD compared with those with the AT + AA genotype and performing moderate/heavy physical activity (OR = 2.80, 95% CI = 1.18–6.64, *p* = 0.020; OR = 3.88, 95% CI = 1.70–8.86), *p* = 0.001). Within the strata of TT, people performing sedentary/light physical activity had a higher risk of CAD than those performing moderate/heavy physical activity (OR = 2.07, 95% CI = 1.14–3.74, *p* = 0.016). People carrying the TT genotype with or without regular fried food intake and those carrying the AT + AA genotype and having regular fried food intake had an increased risk of CAD compared with those carrying the AT+AA genotype and having no fried food intake (OR = 2.38, 95% CI = 1.20–4.74, *p* = 0.013; OR = 3.50, 95% CI = 1.82–6.73, *p* < 0.001; OR = 2.92, 95% CI = 1.44–5.91, *p* = 0.003). People carrying the TT or AT + AA genotype and having regular dessert intake showed a higher risk of CAD than those carrying the AT + AA genotype and having no dessert intake (OR = 2.92, 95% CI = 1.55–5.50, *p* = 0.001; OR = 2.07, 95% CI = 1.04–4.16, *p* = 0.039). The risk of CAD in people with dyslipidemia having the TT genotype and regular dessert intake was 0.85 times higher than that of people with dyslipidemia having the TT genotype and no intake of dessert (OR = 1.85, 95% CI = 1.11–3.07, *p* = 0.018). The interaction between rs4743763 and lifestyle factors was not found in the additive model (*p* values of RERI > 0.05).

Table 4 shows the effects of the interaction between the *ABCA1* rs4149339 and rs4743763 polymorphisms on coronary artery disease. After adjustment for age, sex, waist circumference, smoking, and drinking, compared with people carrying the rs4149339 CT + TT and the rs4743763 AT + AA genotypes, those carrying the rs4149339 CC and rs4743763 TT genotypes demonstrated a higher risk of CAD in dyslipidemia (OR = 4.35, 95% CI = 2.07–9.15, *p* < 0.001). Within the strata of rs4149339 CC, dyslipidemic people with the rs4743763 TT genotype had a higher risk of CAD than those with the AT+AA genotype (OR = 4.66, 95% CI = 1.29–16.79, *p* = 0.019). Within the strata of rs4743763 TT, dyslipidemic people with the rs4149339 CC genotype had a higher risk of CAD than those with the CT + AA genotype (OR = 3.48, 95% CI = 1.71–7.10, *p* = 0.001). A positive interaction between the rs4149339 CC and rs4743763 TT genotypes was found in the additive model (RERI (95% CI) = 3.19 (0.07–6.30), *p* = 0.045).

### 3.4. Association of ABCA1 Haplotypes with CAD Risk 

The haplotype frequencies of the three SNPs were compared between CAD cases and control subjects (Table 5). Five common haplotypes (frequency > 1%) derived from the three SNPs accounting for 95% of the haplotype variation were selected, and the remaining haplotypes were pooled into the rare group. The haplotype CTT was found to be associated with an increased risk of CAD (OR = 1.49, 95% CI = 1.07–2.08, *p* = 0.019).

## 4. Discussion

*ABCA1* is located in the long arm of chromosome 9 (9q31) and is 149 kb long. It is a member of the ATP-binding cassette transporter superfamily, being highly expressed in a variety of tissues and cells such as the liver, intestines, lungs, white blood cells, and macrophages [25,26]. *ABCA1* hydrolyzes ATP, and the energy released is used to transport various molecules across the cell membrane [27]. *ABCA1* is involved in the first step in reversing cholesterol transport by regulating cholesterol and phospholipid efflux from peripheral cells to lipid-deficient apolipoprotein receptors [28,29]. According to both animal experiments and population studies, decreased *ABCA1* activity might result in decreased lipid efflux in peripheral tissue cells. This increases inflammatory signaling factors in atherosclerotic plaques, thereby increasing the risk of coronary artery disease [19,20,22]. In addition, it affects the formation of HDL particles in the liver and intestine [30]. 

The results of this study showed that the CC genotype of the additive model of rs4149339 had a higher risk of CAD compared to the TT genotype; moreover, the recessive model was also significantly associated with CAD. After adjustment for confounding factors the correlation remained. This indicates that the C mutation site of *ABCA1* rs4149339 is a risk factor for CAD in people with dyslipidemia, which is consistent with the results of a case-control study conducted by Lu et al. [31] in China in 2015. The dominant model of rs4743763 was found to be significantly associated with CAD. Even after adjustment for confounding factors, the association remained. This indicates that the T mutation site of *ABCA1* rs4743763 is a risk factor for CAD in dyslipidemia, and it is a newly identified SNP for susceptibility to coronary heart disease in the Chinese population. Meanwhile, an association of ABCA1 rs2472386 with CAD was found in dyslipidemia only in male subjects. This suggests that the association may be influenced by sex.

This study also analyzed the association of physical activity intensity, fried food intake, and dessert intake with CAD in dyslipidemia. These three factors were all found to be significant in the occurrence of CAD with dyslipidemia. Sedentary/light physical activity and the regular intake of fried and dessert increased the risk of CAD in people with dyslipidemia, consistent with most previous studies [32,33,34,35,36]. Physical activity can effectively protect the cardiovascular system from damage. Adequate exercise enhances the myocardial load capacity and increases left ventricular wall thickness and arterial cavity size [37]. Lack of physical activity has been found to result in up to twice the incidence of cardiovascular events, while moderate- to high-intensity physical activity can reduce cardiovascular risk by 10–50% [33]. Sweets and fried foods contain large amounts of saturated and trans fatty acids. In one study, the correlation between saturated fatty acid intake and TC in the blood was found to be 0.23 [38]. If foods with high saturated fatty acid content are used often in daily diets, the plasma levels of TC and LDL-c will increase considerably, and accordingly, the risk of coronary heart disease will increase [39]. After stratification by sex, the association between CAD and behavior in men was consistent with the above, and there was no significant association between the risk of CAD and three behaviors in women, suggesting that we should pay more attention to male subjects when conducting community behavior intervention.

Epidemiological experts believe that quantitative interactions in the additive model are best suited to assess the importance of interaction. In case-control studies, the RERI caused by interaction is generally considered the standard measure of additive model interaction. Our study explored the interactions of *ABCA1* gene-lifestyle factors, SNP–SNP, haplotypes, and certain lifestyle factors with the risk of CAD. RERI, as well as the p values and 95%CI of RERI, are reported herein. Although the interaction index of rs4149339 and rs4743763 with physical activity, fried food intake, and dessert intake was not statistically significant, people carrying risk alleles of rs4149339 and rs4743763 who also performed sedentary/light physical activity or had a regular intake of fried or dessert were also at a high risk for the disease. An interaction between rs4149339 and rs4743763 was found in the additive model. The RERI was 3.19 (95% CI: 0.07–6.30), suggesting that the estimated joint effect on the additive scale of rs4149339 CC and rs4743763 TT genotypes together was greater than the sum of the estimated effects of either genotype alone. Thus, there was a positive interaction on the additive scale. In addition, we found that the haplotype CTT, composed of rs4149339, rs4743763, and rs2472386, might also increase the risk of CAD.

This study had some limitations. Owing to the time limitation of cohort observation, this study only collected cases within a 3 year period, resulting in the inclusion of fewer cases, which might have led to unstable research results. Second, this study was only carried out only in Ningbo. Due to geographical restrictions, the research conclusions might only be applicable to people in southern China. Whether the study findings can be applied to other groups remains to be explored.

## 5. Conclusions

In conclusion, polymorphisms of rs4149339, rs4743763 and rs2472386 in *ABCA1* and three lifestyle factors (physical activity, fried food intake, and dessert intake) were found to be associated with CAD in people with dyslipidemia in southern China. These results provide the theoretical basis for gene screening and the prevention of chronic cardiovascular diseases.

## Figures and Tables

**Table 1:** General characteristics, Lifestyle factors and genotype distribution between case and control groups.

Characteristics	Case	Control	t/z/χ^2^	*p*
	(*n* = 173)	(*n* = 500)		
Age, Median (IQR), y	65 (15)	65 (15)	−0.394	0.693
sex, *n* (%)			0.002	0.963
Male	73 (42.2)	212 (42.4)		
Female	100 (57.8)	288 (57.6)		
Waist circumference, Median (IQR), cm	84 (12)	80 (10.1)	−4.515	**<0.001**
BMI, Mean (SD), kg/m^2^	24.23 ± 3.23	23.11 ± 2.93	−4.210	**<0.001**
SBP, Median (IQR), mmHg	145 (20)	140 (28)	−1.613	0.107
DBP, Median (IQR), mmHg	86 (14)	81 (16)	−3.418	**0.001**
TC, Median (IQR), mmol/L	5.14 (1.31)	5.05 (1.22)	−0.662	0.508
TG, Median (IQR), mmol/L	1.37 (0.88)	1.34 (0.92)	−0.004	0.997
HDL-C, Median (IQR), mmo/L	1.22 (0.4)	1.265 (0.35)	−0.700	0.484
LDL-C, Median (IQR), mmol/L	3.42 (1.06)	3.025 (1.21)	−4.483	**<0.001**
Current smoking, *n* (%)	35 (20.2)	106 (21.2)	0.073	0.787
Current alcohol intake, *n* (%)	43 (24.9)	148 (29.6)	1.423	0.233
Physical activity, *n* (%)				
Sedentary/light	149 (86.1)	360 (72)	13.918	**<0.001**
Moderate/heavy	24 (13.9)	140 (28)		
Fried food intake, *n* (%)				
Never	57 (33.1)	235 (47.1)	10.134	**0.001**
Regular	115 (66.9)	264 (52.9)		
Dessert intake, *n* (%)				
Never	41 (23.7)	182 (36.5)	9.453	**0.002**
Regular	132 (76.3)	317 (63.5)		
rs4149339				
CC	24 (13.90)	37 (7.40)	6.658	**0.036**
CT	67 (38.70)	216 (43.20)		
TT	82 (47.40)	247 (49.40)		
rs4743763				
AA	4 (2.3)	19 (3.8)	4.425	0.109
AT	47 (27.2)	172 (34.4)		
TT	122 (70.5)	309 (61.8)		
rs2472386				
CC	5 (2.9)	20 (4)	2.23	0.328
CT	51 (29.5)	173 (34.6)		
TT	117 (67.3)	307 (61.4)		

IQR, interquartile range; SD, standard deviation; BMI, body mass index; SBP, systolic blood pressure; DBP, diastolic blood pressure; TC, total cholesterol; TG, triglyceride; HDL-C, high-density lipoprotein cholesterol; LDL-C, low-density lipoprotein cholesterol. Bold values are statistically significant with *p* value <0.05 and maintains significance using the Benjamini-Hochberg procedure with the false discovery rate at 0.11.

**Table 2:** Interaction between *ABCA1* rs4149339 polymorphism and lifestyles for the risk of CAD.

Lifestyles	Non-Risk Allele Carriers (CT + TT)		Risk Allele Carriers (CC)		OR (95% CI) for CC Within Strata of Lifestyles	RERI (95% CI)	*p*
	Case/Control (*n*)	OR (95% CI)	Case/Control (*n*)	OR (95% CI)			
physical activity							
Moderately/heavy physical activity	20/134		4/6				
		1		3.83 (0.97–15.06)	3.83 (0.97–15.06)		
				*p* = 0.055	*p* = 0.055		
Sedentary/light physical activity	129/329		20/31			−1.17 (−6.78–4.42)	0.699
		**2.45 (1.46–4.13)**		**4.10 (1.94–8.67)**	1.71 (0.93–3.16)		
		***p*** **= 0.001**		***p*** **< 0.001**	*p* = 0.0860		
OR (95% CI) for Sedentary/light physical activity intensity within strata of genotype		**2.45 (1.46–4.13)**		1.16 (0.22–6.10)			
		***p*** **= 0.001**		*p* = 0.865			
						
fried food intake							
No fried food intake	50/224		7/11				
		1		2.58 (0.94–7.09)	2.58 (0.94–7.09)		
				*p* = 0.067	*p* = 0.067		
Regular fried food intake	98/238		17/26			−0.44 (−3.61–2.73)	0.780
		**1.88 (1.26–2.79)**		**2.98 (1.47–6.02)**	1.62 (0.83–3.18)		
		***p*** **= 0.003**		***p*** **= 0.002**	*p* = 0.161		
OR (95% CI) for regular fried food intake within strata of genotype		**1.88 (1.26–2.79)**		1.22(0.35–4.20)			
		***p*** **= 0.003**		*p* = 0.753			
						
Dessert intake							
No dessert intake	33/74		8/8				
		1		**5.26 (1.81–15.25)**	**5.26 (1.81–15.25)**		
				***p*** **= 0.002**	***p*** **= 0.002**		
Regular dessert intake	116/288		16/29			−3.51(−9.35–2.34)	0.240
		**2.29 (1.47–3.56)**		**3.04 (1.46–6.34)**	1.35 (0.69–2.63)		
		***p*** **< 0.001**		***p*** **= 0.003**	*p* = 0.380		
OR (95% CI) for regular dessert food		**2.29 (1.47–3.56)**		0.51 (0.14–1.79)			
intake within strata of genotype		***p*** **< 0.001**		*p* = 0.290			

RERI, relative excess risk of interaction. Adjusted for age, sex, waist circumference, smoking and drinking. Bold values are statistically with *p*-value < 0.05 and maintains significance using Benjamini-Hochberg procedure with the false discovery rate at 0.11.

**Table 3:** Interaction between *ABCA1* rs4743763 polymorphism and lifestyles for the risk of CAD.

Lifestyles	Non-Risk Allele Carriers (AT + AA)		Risk Allele Carriers (TT)		OR (95% CI) for TT within Strata of Lifestyles	RERI (95% CI)	*p*
	Case/Control (*n*)	OR (95%CI)	Case/Control (*n*)	OR (95%CI)			
Physical activity							
Moderately/heavy physical activity	7/61		17/79				
		1		1.96 (0.76–5.08)	1.96 (0.76–5.08)		
				*p* = 0.167	*p* = 0.167		
Sedentary/light physical activity	44/130		105/230			0.12 (−6.21–6.46)	0.969
		**2.80 (1.18–6.64)**		**3.88 (1.70–8.86)**	1.38 (0.91–2.11)		
		***p*** **= 0.020**		***p*** **= 0.001**	*p* = 0.133		
OR (95% CI) for Sedentary/light physical activity intensity within strata of genotype		**2.80 (1.18–6.64)**		**2.07 (1.14–3.74)**			
		***p*** **= 0.020**		***p*** **= 0.016**			
						
fried food intake							
No fried food intake	13/94		44/141				
		1		**2.38 (1.20–4.74)**	**2.38 (1.20–4.74)**		
				***p*** **= 0.013**	***p*** **= 0.013**		
Regular fried food intake	38/97		77/167			−0.80 (−2.81–1.20)	0.432
		**2.92 (1.44–5.91)**		**3.50 (1.82–6.73)**	1.19 (0.74–1.91)		
		***p*** **= 0.003**		***p*** **< 0.001**	*p* = 0.477		
OR (95% CI) for regular fried food intake within strata of genotype		**2.92 (1.44–5.91)**		1.42 (0.91–2.24)			
		***p*** **= 0.003**		*p* = 0.126			
						
Dessert intake							
No dessert intake	14/80		27/102				
		1		1.59 (0.77–3.27)	1.59 (0.77–3.27)		
				*p* = 0.210	*p* = 0.210		
Regular dessert intake	37/111		95/206			0.25 (−1.09–1.59)	0.711
		**2.07 (1.04–4.16)**		**2.92 (1.55–5.50)**	1.41 (0.89–2.22)		
		***p*** **= 0.039**		***p*** **= 0.001**	*p* = 0.140		
OR (95% CI) for regular dessert		**2.07 (1.04–4.16)**		**1.85 (1.11–3.07)**			
intake within strata of genotype		***p*** **= 0.039**		***p*** **= 0.018**			

Adjusted for age, sex, waist circumference, smoke, drinking. Bold values are statistically with *p*-value < 0.05 and maintains significance using Benjamini-Hochberg procedure with the false discovery rate at 0.11.

**Table 4:** Interaction between *ABCA1* rs4149339 and rs4743763 polymorphism for the risk of CAD.

Gene	Non-Risk Allele Carriers (CT + TT)		Risk Allele Carriers (CC)		OR (95% CI) for rs4149339 CC within Strata of rs4743763 Genotype
	Case/Control (*n*)	OR (95%CI)	Case/Control (*n*)	OR (95%CI)	
Non-risk allele carriers AT + AA	46/172		5/19		
		1		0.83 (0.29–2.38)	0.83 (0.29–2.38)
				*p* = 0.725	*p* = 0.725
Risk allele carriers TT	103/291		19/18		
		1.34 (0.90–2.00)		**4.35 (2.07–9.15)**	**3.48 (1.71–7.10)**
		*p* = 0.161		***p*** **< 0.001**	***p*** **= 0.001**
OR (95% CI) for rs4743763 TT within strata of rs4149339 genotype		1.34 (0.90–2.00)		**4.66 (** **1.29–16.79)**	
		*p* = 0.161		***p*** **= 0.019**	
				

Measure of interaction on additive scale: relative excess risk due to interaction (RERI) (95% CI) =3.19 (0.07–6.30), *p* = 0.045. Adjusted for age, sex, waist circumference, smoking and drinking. Bold values are statistically with *p*-value < 0.05 and maintains significance using Benjamini-Hochberg procedure with the false discovery rate at 0.11.

**Table 5:** Frequencies of haplotypes among cases and controls and association with risk of CAD.

Haplotypes ^a^	Frequency	Cases *n* (%)	Controls *n* (%)	OR (95% CI)	*p*
T T T	0.56	188 (54.2)	563 (56.3)	1	
C A C	0.07	16 (4.6)	69 (6.9)	0.51 (0.23–1.10)	0.086
C T T	0.23	94 (27.3)	212 (21.2)	1.49 (1.07–2.08)	0.019
T A C	0.12	36 (10.4)	129 (12.9)	0.99 (0.62–1.60)	0.990
T T C	0.01	7 (1.9)	11 (1.1)	2.17 (0.81–5.80)	0.121
rare group ^b^	0.02	6 (1.6)	16 (1.6)	0.81 (0.28–2.32)	0.689

^a^ The alleles of haplotypes were arrayed as rs4149339–rs4743763–rs2472386; ^b^ Haplotypes with frequency <0.01 were pooled into the rare group; Adjusted for age, sex, waist circumference, smoke, drink; *p*-value < 0.05 and maintains significance using Benjamini-Hochberg procedure with the false discovery rate at 0.11.

## Supplementary Materials

The following are available online at https://www.mdpi.com/1660-4601/16/5/786/s1, Table S1: Distributions of allele and genotype in the subjects of case and control groups, Table S2: D’value of Linkage disequilibrium for three SNPs of *ABCA*1, Table S3: General characteristics, lifestyle factors and genotype distribution between case and control for male and female, Table S4: Associations of genetic models and lifestyles with risk of CAD for male, Table S5: Associations of genetic models and lifestyles with risk of CAD for female.
