# Supplementary material for: Associations of Genetic Variations in ABCA1 and Lifestyle Factors with Coronary Artery Disease in a Southern Chinese Population with Dyslipidemia: A Nested Case-Control Study
Source: Int J Environ Res Public Health. 2019 Mar 4;16(5):786. doi: 10.3390/ijerph16050786 (PMC6427557; doi:10.3390/ijerph16050786)
Supplement: Supplementary file 1 [file ijerph-16-00786-s001.pdf]

International Journal of Environmental Research and Public Health

MDPI

Supplementary Materials:

Table S1. Distributions of allele and genotype in the subjects of case and control groups.

SNP	A1/A2	MAF	Case	control	HWE-*p* Value
rs4149339	C/T	0.3009	24/67/82	37/216/247	0.3280
rs4743763	A/T	0.1969	4/47/122	19/172/309	0.5001
rs2472386	C/T	0.2036	5/51/117	20/173/307	0.5931

A1: the minor allele. A2: the major allele. MAF: minimum allele frequency.

Table S2. D′ value of Linkage disequilibrium for three SNPs of *ABCA1*.

SNPs	rs4149339	rs4743763	rs2472386
rs4149339		0.108	0.116
rs4743763			0.926
rs2472386			

Table 3. General characteristics, lifestyle factors and genotype distribution between case and control for male and female.

Characteristics		Case (n = 173)	Control (n = 500)	t/z/χ2	p
Age, Median (IQR), y	Male	69(15)	69(15)	−0.163	0.87
	Female	64(16)	63(15)	−0.402	0.688
Waist circumference, Median (IQR), cm	Male	84(12)	80(10)	−2.206	**0.027**
	Female	83(11)	80(11)	−4.057	**＜0.001**
BMI, Mean (SD), kg/m2	Male	23.86 ± 3.22	22.83 ± 3.74	−2.501	**0.013**
	Female	24.49 ± 3.23	23.31 ± 2.88	3.413	**0.001**
SBP, Median (IQR), mmHg	Male	142(20)	140(29)	−0.439	0.660
	Female	145(19)	140(26)	−1.814	0.070
DBP, Median (IQR), mmHg	Male	90(14)	81(16)	−2.477	**0.013**
	Female	86(12)	81(16)	−2.361	**0.018**
TC, Median (IQR), mmol/L	Male	5.09(1.37)	4.99(1.26)	−0.43	0.667
Mean (SD), mmol/L	Female	5.27 ± 1.37	5.19 ± 0.84	0.787	0.432
TG, Median (IQR), mmol/L	Male	1.24(0.89)	1.22(0.81)	−0.231	0.817
	Female	1.44(0.89)	1.48(0.94)	−0.244	0.807
HDL−C,Median(IQR),mmo/L	Male	1.19(0.25)	1.26(0.35)	−2.057	**0.040**
Mean (SD), mmol/L	Female	1.32 ± 0.29	1.29 ± 0.28	0.887	0.376
LDL−C,Median(IQR),mmol/L	Male	3.43(1.15)	2.91(1.18)	−3.816	**＜0.001**
Mean (SD), mmol/L	Female	3.41 ± 0.83	3.17 ± 0.85	2.464	**0.014**
Current smoking, n (%)	Male	34(46.6)	98(46.2)	0.003	0.959
	Female	1(1.0)	8(2.8)	0.399	0.309
Current alcohol intake, n (%)	Male	35(47.9)	116(54.7)	1.000	0.317
	Female	8(8.0)	32(11.1)	0.777	0.378
Physical activity, n (%)					
Sedentary/light	Male	64(87.7)	131(61.8)	16.831	**<0.001**
Moderate/heavy	Male	9(12.3)	81(38.2)		
Sedentary/light	Female	85(85.0)	229(79.5)	1.447	0.229
Moderate/heavy	Female	15(15.0)	59(20.5)		
Fried food intake, n (%)					
Never	Male	20(27.8)	100(47.4)	8.457	**0.004**
Regular	Male	52(72.2)	111(52.6)		
Never	Female	37(37.0)	135(46.9)	2.933	0.087
Regular	Female	63(63.0)	153(53.1)		
Dessert intake, n (%)					
Never	Male	15(20.5)	83(39.3)	8.472	**0.004**
Regular	Male	58(79.5)	128(60.7)		
Never	Female	26(26.0)	99(34.4)	2.384	0.123
Regular	Female	74(74.0)	189(65.6)		

rs4149339						
	CC	Male	35(47.9)	115(54.2)	1.796	0.407
	CT	Male	30(41.1)	83(39.2)		
	TT	Male	8(11.0)	14(6.6)		
	CC	Female	47(47.0)	132(45.8）	6.192	**0.045**
	CT	Female	37(37.0)	133(46.2）		
	TT	Female	16(16.0)	23(8.0)		
rs4743763						
	AA	Male	59(80.8)	120(56.6)	13.636	**0.001**
	AT	Male	13(17.8)	85(40.1)		
	TT	Male	1(1.4)	7(3.3)		
	AA	Female	63(63.0)	189(65.6)	0.682	0.711
	AT	Female	34(34.0)	87(30.2)		
	TT	Female	3(3.0)	12(4.2)		
rs2472386						
	CC	Male	56(76.7)	118(55.7)	10.247	**0.006**
	CT	Male	15(20.5)	86(40.6)		
	TT	Male	2(2.7)	8(3.8)		
	CC	Female	61(61.0)	189(56.6)	1.293	0.524
	CT	Female	36(36.0)	87(30.2)		
	TT	Female	3(3.0)	12(4.2)		

p value <0.05 was considered statistically significant and maintains significance using the Benjamini-Hochberg procedure with the false discovery rate at 0.11.

Table S4. Associations of genetic models and lifestyles with risk of CAS for male.

**SNP/lifestyle**	**genotype/classification**	**Unadjusted OR (95% CI)**	**Unadjusted *p***	**Adjusted OR (95% CI)**	**Adjusted *p***
rs4149339					
additive	TT	1		1	
	CT	1.19(0.68–2.09)	0.550	1.15(0.65–2.03)	0.636
	CC	1.88(0.73–4.84)	0.193	1.92(0.73–5.01)	0.185
dominant	TT	1		1	
	CT+CC	1.29(0.76–2.19)	0.353	1.26(0.73–2.16)	0.405
recessive	CT+TT	1		1	
	CC	1.74(0.70–4.34)	0.234	1.80(0.71–4.57)	0.213
rs4743763					
additive	TT	1		1	
	AT	**0.31(0.16–0.60)**	**0.001**	**0.28(0.14–0.56)**	**＜0.001**
	AA	0.29(0.04–2.42)	0.253	0.30(0.04–2.50)	2.263
dominant	TT	1		1	
	AT+AA	**0.31(0.16–0.59)**	**0.000**	**0.28(0.15–0.55)**	**＜0.001**
recessive	AT+TT	1		1	
	AA	0.41(0.05–3.36)	0.404	0.42(0.05–3.54)	0.427
rs2472386					
additive	TT	1		1	
	CT	0.37(0.20–0.69)	0.002	0.34(0.18–0.65)	0.001
	CC	0.53(0.11–2.56)	0.427	0.58(0.12–2.89)	0.509
dominant	TT	1		1	
	CT+CC	**0.38(0.21–0.70)**	**0.002**	**0.36(0.19–1.67)**	**0.001**
recessive	CT+TT	1		1	
	CC	0.72(0.15–3.46)	0.68	0.81(0.17–3.94)	0.791
Physical activity, *n* (%)	sedentary/light	1		1	
	Moderate/heavy	**0.23(0.11–0.48)**	**0.000**	**0.24(0.11–0.51)**	**＜0.001**
Fried food intake, *n* (%)	Never	1		1	

	Regular	**2.34(1.31–4.19)**	**0.004**	**2.48(1.37–4.51)**	**0.003**
Dessert intake, *n* (%)	Never	1		1	
	Regular	**2.51(1.33–4.71)**	**0.004**	**2.69(1.41–5.12)**	**0.003**

Adjust factors: age, waist, smoking, drinking. p value <0.05 was considered statistically significant and maintains significance using the Benjamini-Hochberg procedure with the false discovery rate at 0.11.

Table S5. Associations of genetic models and lifestyles with risk of CAS for female.

SNP/lifestyle	Genotype/Classification	Unadjusted OR (95% CI)	Unadjusted *p*	Adjusted OR (95% CI)	Adjusted *p*
rs4149339					
additive	TT	1		1	
	CT	0.78(0.48–1.28)	0.327	0.80(0.48–1.34)	0.398
	CC	1.95(0.95–4.01)	0.068	1.90(0.91–3.98)	0.089
dominant	TT	1		1	
	CT+CC	0.95(0.61–1.51)	0.840	0.97(0.61–1.56)	0.905
recessive	CT+TT	1		1	
	CC	**2.20(1.11–4.35)**	**0.024**	**2.11(1.04–4.25)**	**0.037**
rs4743763					
additive	TT	1		1	
	AT	1.17(0.72–1.91)	0.523	1.16(0.70–1.91)	0.568
	AA	0.75(0.21–2.74)	0.664	1.00(0.26–3.83)	0.997
dominant	TT	1		1	
	AT+AA	1.12(0.70–1.80)	0.636	1.14(0.70–1.86)	0.592
recessive	AT+TT	1		1	
	AA	0.71(0.20–2.57)	0.604	0.96(0.25–3.62)	0.949
rs2472386					
additive	TT	1		1	
	CT	1.28(0.79–2.08)	0.314	1.28(0.78–2.11)	0.324
	CC	0.78(0.21–2.84)	0.700	1.10(0.29–4.21)	0.889
dominant	TT	1		1	
	CT+CC	1.22(0.76–1.95)	0.406	1.27(0.78–2.05)	0.337
recessive	CT+TT	1		1	
	CC	0.71(0.20–2.57)	0.604	1.01(0.27–3.84)	0.985
Physical activity, *n* (%)	sedentary/light	1		1	
	Moderate/heavy	0.69(0.37–1.27)	0.231	0.74(0.40–1.40)	0.358
Fried food intake, *n* (%)	Never	1		1	
	Regular	1.50(0.94–2.40)	0.088	1.43(0.88–2.34)	0.154
Dessert intake, n (%)	Never	1		1	
	Regular	1.49(90–2.48)	0.124	1.58(0.93–2.71)	0.092

Adjust factors: age, waist, smoking, drinking. p value <0.05 was considered statistically significant and maintains significance using the Benjamini-Hochberg procedure with the false discovery rate at 0.11.
